# The endocast of the insular and extinct *Sylviornis neocaledoniae* (Aves, Galliformes), reveals insights into its sensory specializations and its twilight ecology

**DOI:** 10.1038/s41598-022-14829-z

**Published:** 2022-12-07

**Authors:** Ségolène Riamon, Jean-Christophe Balouet, Jeanne Rolland-Guillard, Céline Salaviale, Pauline Guenser, Jean-Sébastien Steyer, Antoine Louchart

**Affiliations:** 1 Univ Lyon, UCBL, ENSL, UJM, CNRS, LGL-TPE, 69622 Villeurbanne, France; 2 Environnement International, 60129 Orrouy, France; 3 Université de Bordeaux, CNRS, EPOC, EPHE, UMR 5805, 33600 Pessac, France; 4 Centre de Recherches en Paléontologie – Paris, UMR 7207, CNRS-MNHN-SU, Muséum National d’Histoire Naturelle, 8 Rue Buffon, CP38, 75005 Paris, France

**Keywords:** Palaeontology, Evolution

## Abstract

*Sylviornis neocaledoniae* (Galliformes, Sylviornithidae), a recently extinct bird of New-Caledonia (Galliformes, Sylviornithidae) is the largest galliform that ever lived and one of the most enigmatic birds in the world. Herein, for the first time, we analyze its neuroanatomy that sheds light on its lifestyle, its brain shape and patterns being correlated to neurological functions. Using morphometric methods, we quantified the endocranial morphology of *S. neocaledoniae* and compared it with extinct and extant birds in order to obtain ecological and behavioral information about fossil birds. *Sylviornis neocaledoniae* exhibited reduced optic lobes, a condition also observed in nocturnal taxa endemic to predator-depauperate islands, such as Elephant birds. Functional interpretations suggest that *S. neocaledoniae* possessed a well-developed somatosensorial system and a good sense of smell in addition to its specialized visual ability for low light conditions, presumably for locating its food. We interpret these results as evidence for a crepuscular lifestyle in *S. neocaledoniae*.

## Introduction

Avian evolution on islands has produced some remarkable species exhibiting unique characters and odd morphologies and behaviors, often associated with gigantism: this is particularly the case of the endemic flightless birds such as the iconic dodo (*Raphus cucullatus*) from Mauritius^1^, the impressive moa (Dinornithiformes) from New Zealand^2^ and the giant elephant birds (Aepyornithiformes) from Madagascar^3^. *Sylviornis neocaledoniae* Poplin, 1980, an enigmatic extinct bird from New Caledonia (France), is one such species: the only known representative of its genus, this giant flightless bird (1.3 m in height, 1.7 m in length and 30–35 kg in weight^4,5^) lived on La Grande Terre (the main island of the Archipelago) and L’Île des Pins (small southern island) until the late Holocene^4,5^. It went extinct shortly after the colonization of these islands by humans^4^, which dates to slightly more than 3000 BP^6,7^. Despite extensive osteological studies^4,8^ *S. neocaledoniae* remains enigmatic: vague information is available concerning its overall morphology and behavior has been transmitted thanks to the oral tradition of the autochthonous people^4,9–11^. According to the Kanak tradition, this giant and aggressive bird laid a single egg per year and appeared stealthily during sunset or sunrise^11^: this is the only and unverified “data” on its behavior available so far. The phylogenetic position of *S.* neocaledoniae is the subject of debate^5,8^. Initially thought to be a possible ratite^12^, *S. neocaledoniae* was later considered to be a megapode (Megapodiidae)^4,12^. Later, Mourer-Chauviré & Balouet^8^ erected the family Sylviornithidae for this unique species, based on multiple cranial characters interpreted as autapomorphies. Most analyses have suggested that it is a galliform^5,8,12,13^, possibly a stem galliform^2^, or Galloanserae^5,14,15^. In the latest phylogenetic analyses *S. neocaledoniae* and its sister species *Megavitiornis altirostris* were recovered sister to all galliforms^16,17^. However, its precise phylogenetic position remains to be determined. Moreover, no molecular analysis (e.g., palaeogenomics) has been carried out on sufficiently fresh material. Consequently, *S. neocaledoniae* remains anatomically, ecologically, ethologically and phylogenetically one of the most problematic extinct birds in the world. Here, for the first time we analyze its endocranial anatomy in order to reconstruct its anatomy and behavior. To do so, we compared *S. neocaledoniae* with galliforms and other extant and extinct large birds (Aepyornithiformes, Casuariiformes, Dinornithiformes, Rheiformes, Struthioniformes, Gastornithiformes, Sphenisciformes, Cariamiformes), so as to throw light on its paleoecology. We used non-destructive and non-invasive conventional X-ray computed microtomography (µCT). We targeted internal cranial structures because they are well known to yield precious information about behavior and phylogeny in both avian and non-avian theropods^18,19^. In the absence of the brain endocranial reconstructions are a good alternative to neuroanatomy because, in various extinct and extant birds, there exists a strong correlation and parallelism between the endocranial structures and the underlying brain structures (e.g.,^20–23^): birds generally have a high cranial cavity-brain correlation index (BEC index) resulting from their exceptionally thin meningeal tissues, meaning that inferences regarding sensory capacities can be made^21,24–26^. Moreover, *S. neocaledoniae* is larger than other galliforms. We also consider the size ranges observed in *S. neocaledoniae* because Sayol et al.^27^ showed that cognitive skills in birds are partly related to brain size, which is strongly correlated with body size. This observation stems from the now well-established principle of proper mass, which is used in the study of the brain^25,28^. The relative size of each brain region has also been calculated because it has been showed that it reflects the relative importance of the sensory modalities and specific cognitive processes of the regions of the brain concerned, that is its relative function ^25,29–32^. We are aware that these relative measurements could be biased by allometric factors related to the great size of the animal^25^: in order to minimize this bias, we also compare the endocranium of *S. neocaledoniae* with those of all other large birds (i.e., of body mass greater than 8.750 kg, for the statistical delineation) in addition to extant galliforms. All these comparisons are based on our own 3D-scans realized on various skulls of diverse species (see list in Supplementary Data) and on data extracted from the literature^22,33–36^.

The results of these comparisons are interpreted in terms of sensorial capacities and possible related behavior based on quantitative comparisons of relative cerebral areas.

## Results

### Endocast description and comparison

**Figure 1:**
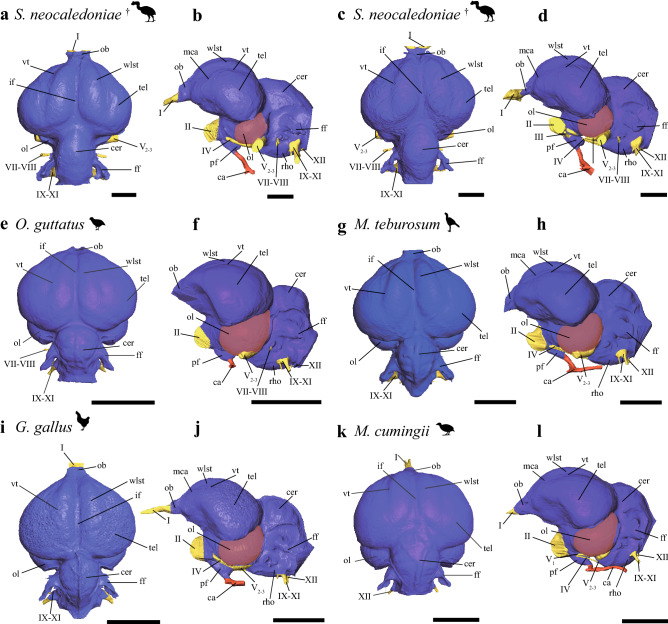
Digital endocranial reconstructions of *Sylviornis neocaledoniae* (**a**, **b**, MNHN NCP 241; **c**, **d**, MNHN unnumbered), *Odontophorus guttatus* (**e**, **f**), *Mitu tuberosum* (**g**, **h**), *Gallus gallus* (**i**, **j**) and *Megapodius cumingii* (**k**, **l**). In dorsal (**a**, **c**, **e**, **g**, **i**, **k**) and left lateral (**b**, **d**, **f**, **h**, **j**, **l**) views. Optic lobes are highlighted in purple to aid in comparison of relative size of this structure across taxa. Colors: blue, endocast; yellow, cranial nerves; red, vasculature. *ca* carotid artery canal, *cer* cerebellum, *ff* floccular fossa, *if* interhemispheric, *mca* medial carotid artery canal, *ob* olfactory bulb, *ol* optic lobe, *pf* pituitary fossa, *rho* rhombencephalon, *tel* telencephalon, *vt* vallecula telencephali, *wslt* wulst, *I* olfactory nerve canal, *II* optic nerve canal, *III* oculomotor nerve canal, *V*_*1*_ ophthalmic nerve canal, *V*_*2–3*_ maxillomandibular nerve canal, *VI* abducens nerve canal, *VII–VIII* facial and vestibulocochlear nerves canal, *IX–XI* glossopharyngeal nerve canal and accessory nerves, *XII* hypoglossal nerve canal. ^†^Fossil taxon. Scale bars = 1 cm.

For ease of reference, the morphological terms specific to the brain used here are defined and described for the different regions of the endocast. The virtual endocasts of two specimens of *S. neocaledoniae* revealed no overall noticeable intraspecific variation (Figs. 1a,c–e) but a morphological similarity to the set of galliforms studied, as opposed to various non-galliform birds, starting from the phylogenetically closest neognaths (see “Materials and methods”) The endocasts of extant galliforms and *S. neocaledoniae* studied revealed interspecific and interfamilial morphological variation (Figs. 1, S3, S4, S5, S7).

### Olfactory bulb

The olfactory bulb, located at the anterior tip of the telencephalon, is composed of two olfactory lobes*. Sylviornis neocaledoniae* shows a slight rostral pinch/tightening of the telencephalon, which delimits the olfactory bulb (Figs. 1, S3–S5). This is also the case in extant galliforms although the shape of their olfactory bulbs varies; individually it is either of the one-lobe type (true fusion of the two bulbs into a single midline bulb) or of the biantennary type (the two bulbs are quite separate)^37–39^. Among *S. neocaledoniae* and studied extant galliforms, the two olfactory lobes are fused as a single lobe but only *S. neocaledoniae* shows an appreciable sulcus delimiting the two lobes, in dorsal view (Fig. S3). The olfactory bulb of *S. neocaledoniae* is narrow and becomes wider at the rostral extremity, from which the olfactory nerves (I) bifurcate. In lateral view, the orientation of the long axes of the olfactory bulbs is different from that of the cerebral hemispheres, as in some galliforms. Unlike Phasianidae and *Odontophorus guttatus* (Odontophoridae), *S. neocaledoniae*, Megapodiidae (*Alectura lathami*, *Megapodius cumingii*) and Cracidae (*Mitu tuberosum*, *Penelope pileata*) have an olfactory bulb located slightly rostroventrally and not in the sub-horizontal position in continuity with the telencephalon. In addition, the olfactory bulb in these groups, has a more ventral inclination (Figs. 1, S4, S5).

### Telencephalon

In dorsal view, the telencephalon of *S. neocaledoniae* exhibits a morphology similar to that of many extant galliforms^22,36,40^: in dorsal and ventral view, the shape of the two cerebral hemispheres gives the rostral part of the endocast a pear-shaped or heart-shaped contour wider caudally. This morphology is much less marked than that of *Acryllium vulturinum*, *Gallus gallus*, *O. guttatus*, *P. pileata* and *Guttera plumifera*. In *S. neocaledoniae*, the mediolateral expansion of the telencephalon begins where the medial cerebral artery traverses the telencephalic hemisphere and in dorsal view, it occludes the rounded optic lobe (Figs. 1, S3). This limit is much less visible in extant galliforms. In dorsal view, this mediolateral expansion in *S. neocaledoniae* is relatively narrower and less caudally developed than that of extant galliforms. In lateral view, the general contour of the telencephalon is sub-rounded, as in *G. gallus* and *Tetrao urogallus*. In other galliforms, the mediolateral expansion of the telencephalon is slightly ventrally pointed, approximately at the rostrodorsal level of the optical lobe. Thus the general contour of the telencephalon is slightly sub-triangular ventrally. In *S. neocaledoniae*, *M. tuberosum* and *T. urogallus*, the mediolateral width of the telencephalon is more developed than the optic lobes. Whereas in other extant galliforms, the mediolateral width of the telencephalon is similar to that of the optic lobes.

Most modern birds have a wulst (sagittal eminence corresponding to the hyperpallium), but it differs in size, shape and position depending on the species^23,41^. The wulst is well defined and delimited dorsolaterally by the vallecula telencephali (Figs. 1, S3). The wulst of *S. neocaledoniae* is relatively developed and as in all galliforms, is in a rostral position (i.e., type A of Walsh and Milner^41^), thus it does not overlap the cerebellum (Figs. 1, S3). In all galliforms, the wulst is delimited from the telencephalon by a shallow groove, the vallecula telencephali. The wulst of *S. neocaledoniae* and that of *M. tuberosum* are more developed than those of other galliforms (Fig. 1). In dorsal view, the wulst of *S. neocaledoniae* is regularly oval in shape, as in *A. vulturinum*, *A. lathami* and *M. cumingii*, whereas in the other galliforms it is dorsolaterally expanded (Fig. S3).

### Diencephalon

The external surface of the endocranial diencephalon is limited to the optic nerve (II) and the pituitary fossa. The optic nerve canal in *S. neocaledoniae* is well developed (Figs. S4, S5). In ventral view, the pituitary fossa is relatively wider in *S. neocaledoniae* and *G. gallus*, than in the other galliforms. The pituitary fossa of *S. neocaledoniae* widens rostrally and it is narrows posteriorly, like all the galliforms studied here, except for *O. guttatus* and *P. pileata*. These have a more rounded pituitary fossa, in lateral view. (Figs. 1, S4)*. Sylviornis neocaledoniae* has a unique morphology of the carotid artery: it remains linked to a point well posterior of the pituitary before subdividing into two distinct branches (Fig. S4). This is not the case in extant galliforms, where the carotid artery is subdivided into two distinct branches at the exit of the pituitary fossa (*G. plumifera, M. cumingii, G. gallus, T. urogallus*) or slightly more posteriorly (*A. lathami, M. tuberosum, A. vulturinum, P. pileata, O. guttatus*) (Figs. 1, S4).

### Mesencephalon

The optic lobes (i.e., the visible parts of the mesencephalon), are located underneath and posterior to the telencephalon. In *S. neocaledoniae* they are somewhat visually inconspicuous structures and seem smaller in comparison to those in the extant galliforms (Fig. 1). They are characterized by a slight lateral expansion of the ventromedial endocast (Fig. S4). In lateral view, the optic lobe of *S. neocaledoniae* is ovoid, as in *T. urogallus*. The other galliforms have a more developed optic lobe rostroventrally (Fig. S4).

### Metencephalon

The morphology of the cerebellum varies individually among the galliforms. The cerebellum of *S. neocaledoniae* is compressed mediolaterally, expanded rostrocaudally, like that of *M. tuberosum* and *G. gallus* (Fig. S3) and it narrows at its rostral and caudal extremities, like that of *T. urogallus* (Fig. S3). In lateral view, the cerebellum of *S. neocaledoniae* is less dorsally domed than that of the other galliforms (Fig. 1). In lateral view, the main axis of the brain of most bird species deviates from the main axis of the spinal cord, resulting a rostral flexion mainly located in the region of the mesencephalon^41,42^ (Fig. 1)*. Sylviornis neocaledoniae* and *T. urogallus* are characterized by the absence of rostral flexion of the cerebellum, reflecting the dorsoventral compression and the elongated shape of their skull.

Among galliforms, the floccular fossa is highly variable in morphology and size. The floccular fossa of *S. neocaledoniae* is parallel to the axial plane and has a lateral projection oriented at 45° angle to the sagittal plane*. Sylviornis neocaledoniae* has a type 5 flocculus (the base is not twisted but is rostrocaudally compressed)^43^. A sheet of bone between the arterial loop and the surface of the fossa is visible: it causes a “fenestra” in the flocculus endocast (Fig. 1). Following Walsh et al.^43^, this structure, covered by an amount of vascular tissue, is characteristic of low manoeuvrability.

### Rhombencephalon

The rhombencephalon forms the caudoventrolateral areas of the hindbrain and defines the structures of the medulla oblongata and pons. In *S. neocaledoniae* and *T. urogallus*, the rhombencephalon is relatively flat rostrocaudally and compressed mediolaterally, unlike those of the other galliforms (Figs. 1, S4). In *S. neocaledoniae*, the cerebellum and associated ventral rhombencephalon form a distinctive hindbrain, which overall morphology differs compared with that of the extant galliforms (Figs. 1, S4).

### Myelencephalon

In *S. neocaledoniae* and all the extant galliforms, the trigeminal ganglion and the base of the trigeminal nerve are contiguous with the optic lobe. This represents the most common condition in extant birds^44^. The trigeminal nerve complex divides into three branches: the ophthalmic nerve (V_1_) and the maxillomandibular complex nerve (V_2–3_) comprising the maxillary nerve (V_2_) and the mandibular nerve (V_3_), and insert on the ventral surface of the optic lobe. The myelencephalon is distinctive in *S. neocaledoniae*, in that the maxillomandibular branch (V_2–3_) is more developed than that of the other galliforms and is practically perpendicular to the ophthalmic nerve (V_1_) (Figs. 1, S4).

### Inner ear

*S. neocaledoniae* and the Cracidae (*M. tuberosum* and *P. pileata*) have more sinuous semi-circular canals than those of other galliforms (Fig. S7). In *S. neocaledoniae*, the semi-circular canals are slightly thicker than those of most galliforms (Fig. S7). Benson et al.^45^ have shown that large non-volant birds have shorter labyrinths*. Sylviornis neocaledoniae* supports this hypothesis in having labyrinths that are proportionately shorter than those of the other galliforms (Fig. S6). A detailed description is given in the electronic supplementary material and is illustrated in Supplementary Fig. S7.

### Endocast orientation

**Figure 2:**
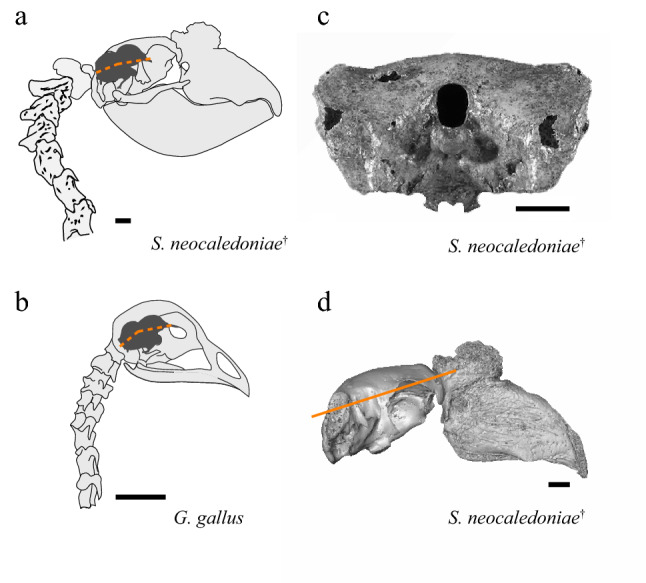
Carriage of the head and orientation of the endocranial axis in *Sylviornis neocaledoniae* (**a**) contrasted with that in *Gallus gallus* (**b**). The endocranial cavity and endocranial axis (orange dashed lines) are superimposed on lateral views of the head and neck in the two species. The attachment of the neck to the back of the skull in *S. neocaledoniae* is accompanied by a dorsally positioned foramen magnum (**c**) and particular orientation of the skull (∽ 15°) relative to the horizontal (orange line, **d**). Scale bars = 2 cm.

Like for the external skull described by Mourer-Chauviré and Balouet^8^ (and Fig. 2c), the endocast of *S. neocaledoniae* does not show a great dorsoventral expansion. When the skull is oriented in a normal position, that is following the horizontal of the lateral semi-circular canal, the reconstructions show that the flexion (the endocranial axis from the tip of the olfactory bulb to the foramen magnum through the midpoint of the isthmic constriction) of the endocranial cavity of the two *S. neocaledoniae* specimens was significantly lower (averaged 17.7°) than that of the other galliforms examined (averaged 30.9°) (Fig. S10). This low brain flexion of *S. neocaledoniae* is associated with a particular orientation of the skull (∽ 15°) relative to the horizontal (Fig. 2). These two observations are consistent with a horizontal posture/position of the upper neck as in the more recent skeletal reconstructions^2^. This horizontal posture and its effect on the orientation of the endocast and the relatively dorsal position of the foramen magnum are illustrated in Fig. 2. These observations support the hypothesis of a correlation between skull size, brain orientation, foramen magnum position and posture of the head in birds^46,47^. Most endocasts of adult neognath birds are steeply inclined upwards, ventrally flexed or narrower and more anteriorly inclined^30^ whereas in *S. neocaledoniae* it is narrower and weakly inclined downwards. Ashwell and Scofield^48^ showed that most moa also tend to have low values for endocast flexion, induced by horizontal deportment from their upper neck. The entire endocast has a relatively elongated general morphology like that of *T. urogallus*.

### Size of endocast structures

#### Olfactory bulb size

**Figure 3:**
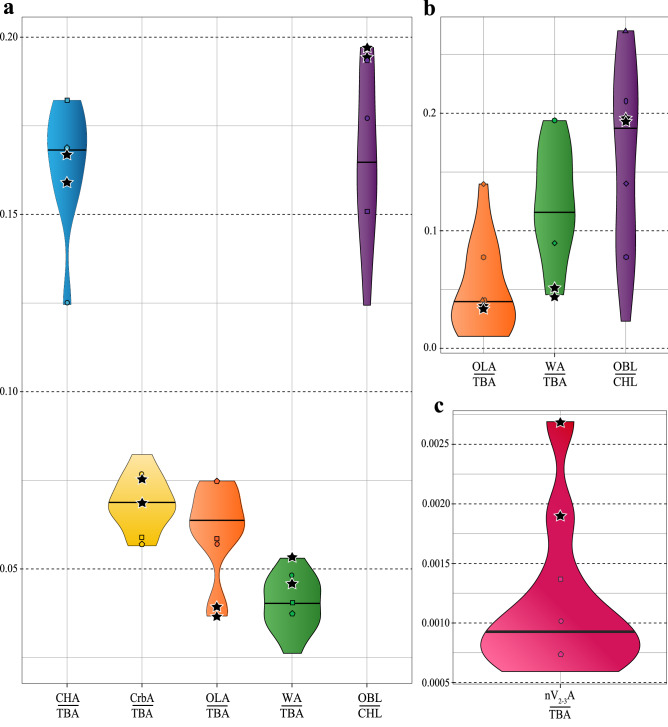
Violin plots showing the position of *Sylviornis neocaledoniae* (black star) relative to other Galliformes, with n = 11 (**a**, **c**) and large birds (body mass greater than 8.750 kg), with n = 9 for the ratio of wulst and n = 15 for the two other ratios (**b**), according to a series of ratios. Heavy black line indicates the median. Distribution of certain species: circle, *Tetrao urogallus*; square, *Mitu tuberosum*; pentagon, *Odontophorus guttatus*; rhombus, *Meleagris gallopavo*; triangle, *Casuarius casuarius*; hexagon, *Llallawavis scagliai*; ellipce, *Pachyornis elephantopus*. *CHA* cerebral hemisphere surface area, *CHL* cerebral hemisphere length, *CrbA* cerebellum surface area, *OLA* optic lobe surface area, *OBL* olfactory bulb length, *nV*_*2–3*_*A* maxillomandibular nerve canal cross-section area, *TBA* total brain surface area, *WA* wulst surface area.

The relative development of the olfactory bulb in *S. neocaledoniae* is the largest of all galliforms and positions itself close to *O. guttatus* and *T. urogallus* (Fig. 3, Tables S1, S3). When the ratio of olfactory bulb is compared to that of other large birds, it is positioned close to the median: *S. neocaledoniae* has an olfactory bulb almost as developed proportionately as in the giant moa (*Pachyornis elephantopus*) and more developed than that of the ostrich (*Struthio camelus*) (Tables S1, S3). In birds, the relative size of the olfactory bulb is correlated with olfactory capacity^28,38,49^*. Sylviornis neocaledoniae* therefore probably had strong olfactory abilities and the best sense of smell among galliforms measured here.

#### Optic lobe size

In contrast, the optic lobes of *S. neocaledoniae* are relatively the smallest of all galliforms (Fig. 3a, Tables S1, S3). When the relative size of the optic lobe is compared with that of all birds or large birds^33,34,50,51^, it is positioned close to the medians and means in both cases (Fig. 3b, Tables S1, S3): *S. neocaledoniae* has an optic lobe as reduced as that of the cassowary (*Casuarius casuarius,* Casuariidae), an extinct penguin (*Paraptenodytes antarcticus,* Spheniscidae) and the magnificent bird of Scaglia (*Llallawavis scagliai*, Phorusrhacidae). In birds, the relative size of the optic lobe is correlated with optical capacity^22,33,50,51^*. Sylviornis neocaledoniae* therefore probably had a weak visual acuity, as is also suggested by its proportionately reduced optic nerve and its small orbits (see electronic supplementary material, Fig. S11).

#### Wulst size

Relative to endocast, the wulst of *S. neocaledoniae* is one of the largest among galliforms, with *T. urogallus* and *Meleagris gallopavo* (Fig. 3a, Tables S1, S3). However, when the relative size of this wulst is compared to that of large birds^34,50,51^, it is one of the smallest wulsts of large birds (Fig. 3b, Table S3).

#### Maxillomandibular nerve (V_2–3_) section

The relative size of the maxillomandibular nerve canal cross-section (V_2-3_), provides information about the development of the somatosensory system (sense of touch) and presumably mechanoreception. The comparison does not include large birds because of the lack of data. In galliforms, the maxillomandibular nerve is weakly developed in general (average ratio of: 0.0097 against 0.023 for *S. neocaledoniae*)*. Sylviornis neocaledoniae* presents a much more developed nerve than that of extant galliforms (Fig. 3c, Tables S1, S3), except for that of *Mitu tuberosum* (Tables S1, S3). Apart from *S. neocaledoniae* another Galloanserae shows an exceptionally well developed maxillomandibular nerve: the extinct anseriform *Talpanas lippa* of the Hawaiian Islands (Mole-duck, Oxyurini)^52,53^ (Fig. S11)*. Sylviornis neocaledoniae* shows a relatively smaller maxillomandibular nerve than *T. lippa* proportional to the size of the endocast, but slightly more developed compared to the surface of the foramen magnum (Fig. S11).

## Discussion

The endocranium of *S. neocaledoniae* is similar to that of a typical galliform, but, like its general osteology, it also shows a mosaic of derived characters, galliform synapomorphies and probably some plesiomorphies. A precise phylogenetic analysis of *S. neocaledoniae* is outside the scope of this study, however, the cranial and endocranial anatomy of *S. neocaledoniae* reveals very interesting traits concerning its behaviour and lifestyle:

The shape and orientation of the endocast is caused in part by the foramen magnum being located caudally and facing caudally on the cranium, and it is not directed somewhat ventrally as in most birds. The fact that its cerebellum has no rostral flexion is linked to the general shape and orientation of the endocast, and the high dorso-caudal position of the foramen magnum, also observed exclusively in some moa^48^ and a giant terror bird^54^. Gussekloo and Cubo^55^ have shown that the inability to fly in modern birds affects the morphology of the skull. They suggest that this relationship is related to a reduced selective force for light skull in these birds, allowing the development of strong muscle insertions of a heavier skull and robust neck in walking birds that are unable to fly. These authors confirmed the relationship between flight incapacity and a peramorphic (i.e., overdeveloped) skull in relation to the standard ontogenetic trajectory of *G. gallus*, suggested by morphological studies^56^. We can assume that the cranial morphology of *S. neocaledoniae* follows this same trend (e.g. see the morphology of the palatine-pterygoid complex^8^), but a more detailed morphological and quantitative study is needed to test this hypothesis. Other parts of the skeleton such as the reduced keel and wings are typically interpretable as paedomorphic, and such a mosaic of different heterochronic conditions is observed in other flightless birds^57^.

The relative importance of sensory modalities and specific cognitive processes is reflected in the relative sizes of brain regions^25,29–31^. Our reconstructions of the *S. neocaledoniae* endocasts, compared with both extant galliforms and extant and extinct “large” birds (i.e., above 8.570 kg), show that this bird had relatively small optic lobes associated with small orbits and optic nerves (Figs. 3, S11). The visual information is processed along three different pathways in the avian brain: accessory optic, thalamofugal, and tectofugal pathways of which the last two are most important and different. The optic lobe is the equivalent of the visible part of the optic tectum and optic tract and is the relay point for sensory information to the anterior brain^22,58^. The optic lobe comprises most of retinal afferents, is related to retinal summation and hence acuity and corresponds to the main visual pathway in birds^59–61^. Early et al.^22^ showed that the relative size of the optic lobes is representative of the size of the underlying brain structure. Thus we supposed that behavioral inferences can be made from brain-structure size. Among extant birds, relatively small optic lobes are observed only in crepuscular or nocturnal birds such as the kiwi^62^, the nocturnal parrot *Pezoporus occidentalis*^63^, and the cassowary^33^. Optic lobe reductions in the elephant birds and the Mole duck *Talpanas lippa*, have been interpreted as revealing a nocturnal lifestyle pattern for these extinct birds^33,52,64^. This correlation is consistent with the proper mass principle^25^, which predicts that the relative development of a brain structure is proportional to the complexity of the associated behaviours, which is confirmed by studies on extant species^22,33,38,41,62,64^. Therefore, as evidenced by the small optic nerves and lobes associated with the small orbits, the visual system of *S. neocaledoniae* implies reduced visual performance. Compared to the extant galliforms, *S. neocaledoniae* did not have a good ability to discern information brought to the brain by sight and thus poor vision. Biometrically, *S. neocaledoniae* exhibits endocranial ratios similar to those of the cassowary^33^ and, among the galliforms studied, it has the smallest relative surface of the optic lobes (Fig. 3, Tables S1, S3). Additionally, *S. neocaledoniae* also has an optical lobe ratio (to the endocast area) similar to that of the diurnal emu (*Dromaius novaehollandiae*), as given in the Torres & Clarke^33^ study, but is lower than that given for the emu in Corfield et al.^51^ (Table S3). This assumes some relatively large intra-species variation in emu for ratios of optic lobe and the other different brain structures (Table S3), or more likely differences between how different workers measured these data. The cassowary has a greater activity at the beginning and the end of the day, which corresponds to a crepuscular lifestyle. Assuming that the correlation between this structural reduction and this lifestyle is valid for *S. neocaledoniae*, then it is inferred that it had reduced visual acuity and probably a crepuscular lifestyle.

To respond to the transition from a mainly diurnal towards a crepuscular or nocturnal lifestyle, the bird’s visual system can either increase its visual sensitivity or reduce it in favour of other senses^62^. Increased sensitivity characterizes flying nocturnal birds (e.g., owls, oil birds, nightjars), which specialize in manoeuvrability and feeding in low light conditions, often characterized by large eyes and hyperdeveloped wulsts^65^. Only nocturnal flightless insular birds are known to have reduced the visual system in favour of other senses, including the Malagasy elephant birds, New Zealand moa, and the kakapo^33,62,66,67^. However, the last species appears to have atypical eye anatomy for diurnal or nocturnal activity^64^. The relatively small size of the eyes and optical lobe in the Moa, have been recently interpreted in terms of a nocturnal ecology for these giant flightless birds from New Zealand^33^. However, the study of *Megalapteryx didinus* (Dinornithiformes); the only species for which sclerotic rings are known^64^, indicated cathemeral (even diurnal/nocturnal) activity.

The adaptation of *S. neocaledoniae* to a crepuscular activity corroborates the hypothesis of Torres and Clarke^33^, in which reduced vision in birds (that are nocturnal or crepuscular and which use their other senses) is an adaptation that is probably an option only for flightless species (or really advanced only in those species). This hypothesis is based on the fact that despite the observation that a visual capacity that is adapted to low light conditions exists in various clades across many terrestrial non-avian vertebrates, only birds exhibit a correlation with changes in locomotion strategy, that is, loss of flight. But this might be easily explained by the correlation between loss of flight, living in predator-free or predator-scarce, insular environments, and viability of reduced vision. Indeed, high-performance vision is rendered less indispensable as soon as there are no more flight requirements (manoeuvrability etc.) nor any (or reduced) need to escape predators.

It can be hypothesized that vision capacities have decreased in favour of the use of other senses, such as smell, touch or hearing^62,68,69^. These non-visual senses may also have been used by *S. neocaledoniae* in adapting to a crepuscular lifestyle. The relatively large size of the olfactory bulb of *S. neocaledoniae* seems to attest to a well-developed sense of smell. Zelenitsky et al.^28^ showed that the olfactory bulb ratio and body mass are uncorrelated in birds. However, they observed that in large species of Neornithes (more than 4.85 kg, in Zelenitsky et al.^28^), the olfactory ratios tend to be lower than those predicted by the regression. *Sylviornis neocaledoniae*, being a large bird, has a smaller olfactory bulb ratio than predicted by this regression and so confirms this tendency. However, it has proportionately the largest olfactory bulb of all galliforms and its ratio is larger than average, among large birds. Therefore, we consider that *S. neocaledoniae* had a relatively well-developed sense of smell. This well-developed sense of olfaction was perhaps crucial while foraging or was correlated with a particular, closed, forested habitat in which vision (and even hearing) were hampered (as Torres and Clarke^33^, have hypothesized with elephant birds).

For some species, the adoption of a crepuscular or nocturnal lifestyle may lead to a development of the second visual pathway; the thalamofugal pathway, which projects from the retina to the dorsal thalamus and onto the wulst^70^, resulting in a relatively enlarged wulst. This peculiarity is present and well known in Strigiformes especially, unlike kiwi (Apterygiformes) that lack a developed wulst. However, some diurnal species also possess a relatively enlarged wulst, e.g., emus, and therefore probably had a developed thalamofugal visual pathway. The thalamofugal pathway is devoted to integrating visual information^71^, for example, associated with stereopsis^65^, spatial orientation^72^, pattern discrimination at a distance^73^. Nocturnal or crepuscular taxa therefore require an increased sensitivity of the visual system, especially in low light environments^65,70^*. Sylviornis neocaledoniae* seems to have a relatively well-developed wulst (among all birds, it is the best-developed within galliforms). Therefore, its stereopsis was apparently developed. Early et al.^34^ speculated that the position of the wulst is correlated with the use of the tactile sense when feeding. Birds with a wulst in the caudal position (type B sensu Walsh & Milner^41^), use the tactile sense while foraging, unlike birds with a wulst of type A (in rostral position). We suppose that the latter have a developed sense of touch that is not used for foraging but could help to understand and perceive their environment, thus compensating for the possible reduction of other senses. As *T. lippa* among anseriforms, *S. neocaledoniae* also has a maxillomandibular nerve that is proportionately wider than in the extant galliforms examined. However, it should be noted that the enlargement in *T. lippa* was extraordinary, and enlargement is not so great in *S. neocaledoniae.* This nerve is directly related to the somatosensory system (the perception of touch, temperature, body position (proprioception), and pain) of the beak*. Sylviornis neocaledoniae* would therefore have had a well-developed somatosensory system of the beak to compensate for its reduced visual acuity. This developed sense agrees with the large size of its beak, its vascularization and the presence of numerous foramens at the rostral extremity of the beak^8^. This sense may have served it to investigate its environment, using its beak as a tester. It is difficult to be more precise, but if there were Herbst receptors associated to the numerous foramens at the rostral extremity of the beak, such a tactile sense could correspond to foraging in the soil, the humus of the forested habitats, or under leaves. Enhanced beak somatosensory capacities could also relate to enhanced mechanoreception, possibly in relation to special use of the beak that might have exerted particular mechanical constraints. The maxillomandibular nerve could relate to the presence of pressure receptors for skillful manipulation of food. The mechanoreceptors are just one of the many potential things that this nerve transmits, and it can be bound to taste receptors. Indeed, the gustative information from the tongue is conveyed, within the lingual branch of the maxillomandibular (V_2-3_) ramus, by the facial (VII) nerve to the trigeminal principal sensory nucleus, which also receives input from glossopharyngeal (IX) and hypoglossal (XII) nerves^36^. But we consider it to be quite possible that the beak, with its peculiar morphological characteristics^8^ of a greatly enlarged bony casque dorsally, was used for other and still unknown purpose(s) (mate selection for example?). Further analyses will be carried out to try to answer these questions.

The ecology of *S. neocaledoniae* seems to have been highly peculiar, and more complex than thought earlier. The adoption of a crepuscular lifestyle by *S. neocaledoniae* seems to be correlated to a reduction in visual acuity and specialised visual ability for low light conditions, associated to the development of smell and somatosensory system of the beak, linked to the loss of flight permitted by the absence of predators in an oceanic insular environment. Moreover, *S. neocaledoniae* seems to have substantially developed its somatosensory system, the only other such example known being the extinct, highly peculiar Hawaiian Mole duck *T. lippa*^52^*. Sylviornis neocaledoniae* stands out as one of the most eccentric recently extinct bird species known, highlighting the fact that the most divergent species, morphologically, ecologically and behaviourally speaking, went preferentially extinct as a result of the arrival of humans on the islands where it lived, and that such a threat is still extremely great for the surviving insular endemics all over the world^74^.

## Materials and methods

### Endocast reconstruction

We reconstructed two digital endocasts of *S. neocaledoniae* with high-resolution X-ray computed microtomographic methodology, based on two well-preserved cranial specimens from the collections of the Muséum National d’Histoire Naturelle (Paris, France); MNHN-NCP 241 and an MNHN-NCP unnumbered). For our comparative analysis, we also reconstructed the endocrania of at least one representative of all extant galliform families, one extant species of anseriform (the extant sister group of galliforms^75,76^) and representatives of other orders: Phoenicopteriformes, Podicipediformes, Gruiformes, Charadriiformes and Opisthocomiformes. Virtual endocasts were reconstructed using Avizo 9 lite (FEI Visualization Sciences Group, Berlin, Germany), following the reconstruction suggestions of Balanoff et al.^21^. Only practices to smooth and connect the separate portions of the endocasts were used. Scanning parameters and details of methods are provided in the electronic supplementary material (see Table S2). For all specimens, skull, endocast, base of the major nerves and labyrinths (anterior (ASC), lateral (LSC) and posterior (PSC) semi-circular canals, vestibule and cochlear canal) were segmented. Data for other species are derived from other studies^22,33,36,50,51,77^ and their data and endocast reconstructions contributed to this study (see Tables S1, S3).

### Measurements of the olfactory bulb, optic lobe, and semicircular canals

The olfactory bulb of extant species has been difficult to reconstruct, because its surrounding is not completely ossified. In some species, the olfactory nerves could not be reconstructed and the lower limit of the olfactory bulb is not always well defined. We were therefore unable to carry out surface measurements for the olfactory bulb. The measurements of the olfactory bulb were therefore carried out according to the method described of Bang and Cobb^38^: the ratio of length of the olfactory bulb over the length of the corresponding cerebral hemisphere, and in the axis of the maximum length for each measure. To investigate olfactory bulb relative size across galliforms and other birds, this ratio was then compared with the species sampled by Bang and Cobb^38^, Torres and Clarke^33^ and Zelenitsky et al.^28^.

The measurement of the optic lobe follows Torres and Clark^33^ but we used the ratio of the surface of the two optic lobes (and not a single optic lobe) on the total surface of the endocast. The measurement was carried out with the MeshLab software version 2020.06. Detailed methods of acquiring surface measurements are described in the electronic supplementary material and illustrated in Supplementary Fig. S1.

We measured the relative positions of the semi-circular canals (i.e., the angles between the canals) and their length using Avizo 9 lite (FEI Visualization Sciences Group, Berlin, Germany). The measurements follow Benson et al.^45^. All measurement data are provided in the electronic supplementary material (Table S1). Detailed methods are described in the electronic supplementary material and illustrated in Supplementary Fig. S2.

### Nomenclature

We follow the anatomical nomenclature in Baumel and Witmer^78^, for osteology, external brain surface structure and innervation. Disparate terminologies have been used for the description of the surface morphology of the avian cranium and brain, in some instances with no consensus for any particular term (e.g.,^78,79^). The endocasts have been described in accordance with the terminology of Witmer et al.^18^, Early et al.^23^ and Handley and Worthy^36^, and are illustrated in the Figs. 1, S1, S3, S4 and S5.

## Supplementary Information

Supplementary Information 1.

Supplementary Table S1.

## Data Availability

The original X-ray microtomography files for the specimens in this study are available on request and will be uploaded to MorphoSource.
