# Supplementary material for: The endocast of the insular and extinct Sylviornis neocaledoniae (Aves, Galliformes), reveals insights into its sensory specializations and its twilight ecology
Source: Sci Rep. 2022 Dec 7;12:21185. doi: 10.1038/s41598-022-14829-z (PMC9729198; doi:10.1038/s41598-022-14829-z)
Supplement: Supplementary file 1 — Supplementary Information 1. [file 41598_2022_14829_MOESM1_ESM.pdf]

Electronic Supplementary Material

The endocast of the insular and extinct bird Sylviornis neocaledoniae (Aves, Galliformes) reveals insights into its sensory specializations and its twilight ecology.

Ségolène Riamon, Jean-Christophe Balouet, Jeanne Rolland-Guillard, Céline Salaviale, Pauline Guenser, Jean-Sébastien Steyer, Antoine Louchart

This PDF file includes :

Methods
Supplementary Figures
Descriptions
Supplementary Tables
References

Methods

Sampling and scanning

Computed tomography data sets were acquired with the Phoenix Nanotom S micro-tomograph belonging to the Lyon Gerland Federal Research Structure (SFR Biosciences UMS3444/US8), located on the premises of the Ecole Normale Supérieure de Lyon (ENS). Scanning parameters are listed in Table S2. All the specimens were acquired, for this study, with X-ray power equal 100/0.07 kV/mA. The endocast of the chicken (*Gallus gallus*: NMS-Z.1931.43; MorphoSource ARK: ark:/87602/m4/M33493) was segmented from data from Benson et al., 2017 available on MorphoSource (http://morphosource.org/Detail/ProjectDetail/Show/project_id/377). We reconstructed the endocasts of nine species Galliformes in addition to two endocasts of *S. neocaledoniae* (an uncatalogued MNHN specimen and MNHN-NCP 241). We selected our sample so that each galliform family is represented by one or more species. This sample consists of two megapods (*Alectura lathami lathami*: MNHN-1923-2001 and *Megapodius cumingii*: LGL-1.177), a guan (*Penelope pileata*: LGL-1.396), a curassow (*Mitu tuberosum*: LGL-1.694), two guineafowls (*Guttera plumifera*: LGL-782 and *Acryllium vulturinum*: LGL-1.391), a wood quail (*Odontophorus guttatus*: LGL-1693), and a capercaillie (*Tetrao urogallus*: MNHN-ME499). To complete this study, we also scanned a specimen of Anseriformes, Phoenicopteriformes, Podicipediformes, Gruiformes, Charadriiformes and Opisthocomiformes (Table S2), to provide a phylogenetically framework the clade of Galliformes (Kimball et al. 2019; Kuhl et al. 2021).

Model Reconstructions

The scan data was processed by the 3D visualization program Avizo 9 lite (FEI Visualization Sciences Group, Berlin, Germany). In Avizo, using the program’s “magic wand”, “paint” and “select” tools, the voxels corresponding to the different endocast spaces were isolated and assigned to materials respectively (Balanoff et al., 2016). The endocast of the specimen of interest corresponds to the production of this virtual model. As with some previous studies (e.g. Early, 2019; Early et al., 2020a; Torres & Clarke, 2018), during the segmentation of endocasts, particular attention was paid to the removal of the vascular and nervous system of the endocast along the boundaries where these structures intersect each other. The boundary between the vascular or nervous system and the endocast, was guided by the points of flexure between these structures and the endocast, in the three different views. Due to its diffuse limits, only the

occipital sinus was not removed in any taxa. These removals were performed as consistently as possible to ensure that the vascular and nervous system did not alter the estimates of the overall endocast area and volume.

Measurements

Measurements of surfaces of cerebral regions areas of the endocast were taken using Meshlab software version 2020.06 (Cignoni et al., 2008). The volume and total endocast area were measured using the "Compute Geometric Measure" tool. The surfaces of the different structures of the endocasts were obtained by using the "Z-painting" tool selection brush to select the area of interest, then the "Compute Area of selection" tool. The surfaces measured are illustrated in Supplementary Figure S1 and have been delineated as follows: (i) the cerebral hemisphere includes the occipital, parietal, and frontal parts of the telencephala telecula as well as the ventromedial and ventrolateral tubercles previously, and is dorsally delimited by the vallecula telencephali, following Baumel (1993, p527). (ii) The Wulst or sagittal eminence is bounded by the vallecula telencephali and the interhemispheric fissure. (iii) Optic lobe measurement excludes trigeminal nerve and semi-circular vein. (iv) The limits of the cerebellum are the posterior sill to the telencephalon, without the pineal gland if any, the semi-circular veins and the dorsal edge of the foramen magnum. Measurements made on the inner ear follow Benson et al. 2017: (i) length of the cochlear duct, in successive segments to follow the curve, (ii) lengths of the three semi-circular canals, in small successive segments, the tubular part of the canal between their ampulla and the crus communis for the anterior (ASC) and posterior (PSC) canals and the vestibule for the lateral canal (LSC), and (iii) angles between pairs of semi-circular canals (Figure S2).

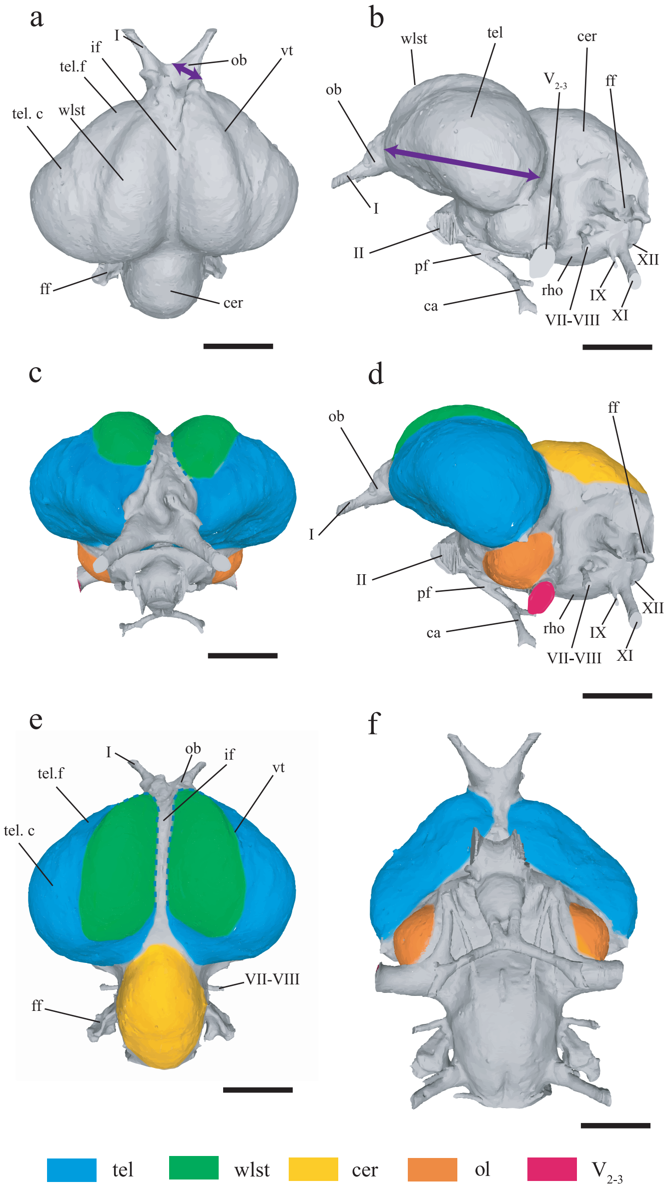

Figure S1. Illustration of linear measurements (a, b) and surface measurements (c, d, e, f) on *S. neocaledoniae* endocast. The areas measured are: cerebral hemisphere area (blue); wulst area (green); optical lobe area (orange); cerebellum area (yellow) and maxillomandibular nerve section area (pink). a, the double arrow indicates the length of the olfactory bulb (longest axis); b, the double arrow indicates the length of the cerebral hemisphere (longest axis). The dotted lines (blue) show the measured area of the cerebral hemisphere, including the wulst (c, e). Abbreviations: ca, carotid artery canal; cer, cerebellum; ff, flocculus if, interhemispheric fissure; ol, optic lobe; ob, olfactory bulb; pf; pituitary fossa; rho, rhombencephalon; tel, telencephalon; tel.f, frontal telencephalon; tel.c; caudal telencephalon; vt, vallecula telencephalic; wslt, wulst; I, olfactory nerve canal; II, optic nerve canal; V_{2-3}, maxillomandibular nerve canal; VII-VIII; facial and vestibulocochlear nerves canal; IX, glossopharyngeal nerve canal; XI; accessory nerves; XII, Hypoglossal nerve canal. (a) sub-dorsal view; (b, d) lateral view; (c) rostral view; (e) dorsal view; (f) ventral view. Scale bars = 1cm

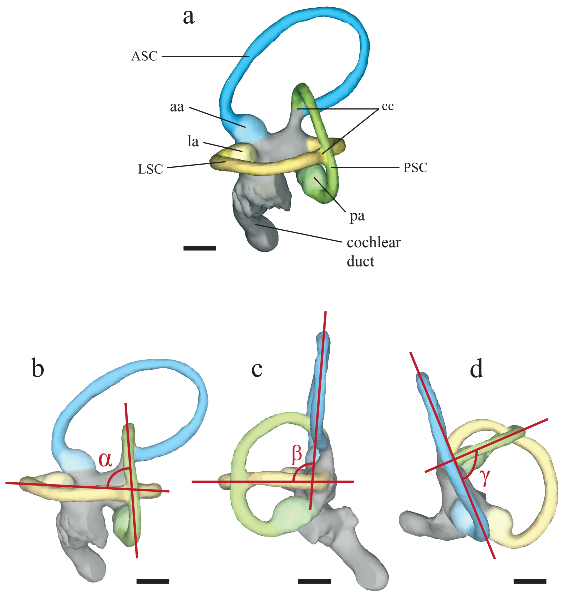

Figure S2. Process of segmenting (a) and measures of angles between semi-circular canals (b, c, d) the avian labyrinth. *Gallus gallus*, NMS:Z.1931.43 (a, b, c, d) Abbreviations, aa, ampulla of the anterior semi-circular canal (light blue); ASC, anterior semi-circular canal (blue); cc, crus communis; la, ampulla of the lateral semi-circular canal (light yellow); LSC lateral semi-circular canal (yellow); pa, ampulla of the posterior semi-circular canal (light green); PSC, posterior semi-circular canal (green). Scale bars = 2mm.

Descriptions

Innervation

Olfactory nerve

The olfactory nerve canal (I) transmits rostro-ventrally into the olfactory bulb, through the n. olfactorii (Figures 1, S3, S4, S5). The *S. neocaledoniae* olfactory nerves canals are very well delimited by the bony foramen, unlike the other galliforms studied whose olfactory nerves canals and olfactory bulb have only dorsal bony limits (Figures S3, S4). The *S. neocaledoniae* olfactory nerves canals are rostrally divergent while those of the galliforms studied are parallel (Figures 1, S3, S4, S5).

Optic nerve

The same as for the olfactory nerve, the optic nerve (II) is poorly delineated in galliforms, the interorbital spetum being little or no ossified. Unlike the Galliformes in *S. neocaledoniae*, the optic nerves are divided rostrolaterally into two distinct branches (Figures 1, S4, S5), and are well developed.

Nervus abducens

As in the Dromornithidae (Handley & Worthy, 2021), the *S. neocaledoniae* nerve abducens (VI) is paired with the nerve ophthalmic (V_1) (Figure S4) thus forming only one bi-lobal foramen. This character is also present in some Galliformes: *G. plumifera, Acryllium vulturinum* and *Tetrao urogallus*.

Nervus Glossopharyngeus

In *S. neocaledoniae*, the separation between the glossopharyngeus nerve (IX) and vagus (X) is similar to that of other Galliformes but occurs more distally (Figures 1, S4).

Nervus Hypoglossus

The nervus hypoglossus (XII) of *S. neocaledoniae* are represented by one ramus at either side of the caudoventrolateral medulla oblongata, and not by the typical rostral (XIIr) and caudal (XIIc) rami. This character seems to be present in all galliforms studied here.

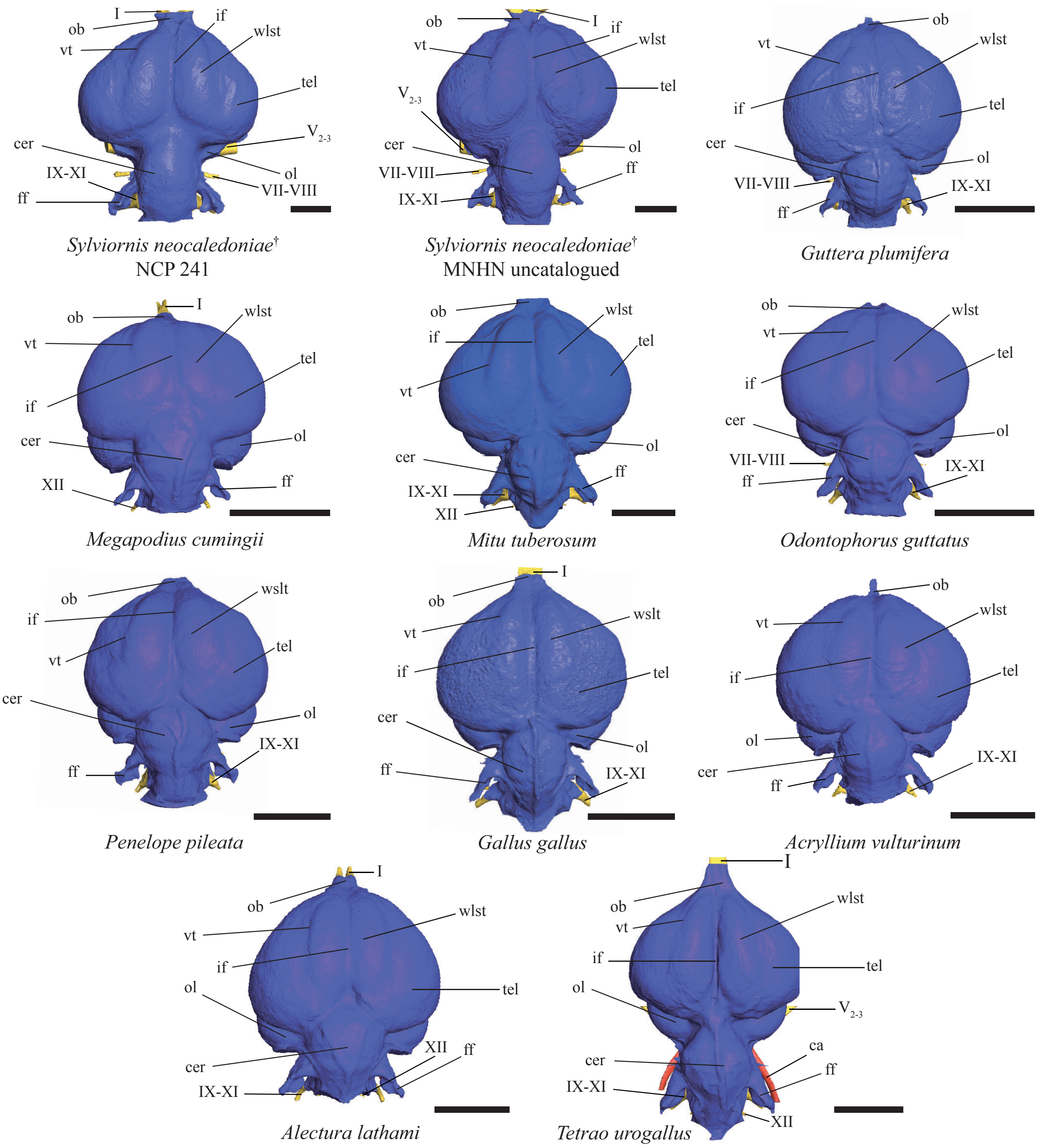

Figure S3. Dorsal view of *S. neocaledoniae* and Galliformes endocasts. *Guttera plumifera* (Numididae, FSL-782); *Megapodius cumingii* (Megapodiidae, FSL-1.177); Mitu tuberosum (Cracidae, FSL-1.694); *Odontophorus guttatus* (Odontophoridae, FSL-1693); *Penelope pileata* (Cracidae, FSL-1.396); *Gallus gallus* (Phasianidae, NMS:Z.1931.43); *Acryllium vulturinum* (Numididae, FSL-1.391); *Alectura lathami* (Megapodiidae, MNHN-1923-2001); *Tetrao urogallus* (Phasianidae, MNHN-ME499). Abbreviations: ca, carotid artery canal; cer, ol, optic lobe; tel, telencephalon; vt, vallecula telencephalic; wslt, wulst; I, olfactory nerve canal; $V_{2\text{-}3}$, maxillomandibular nerve canal; VII-VIII; facial and vestibulocochlear nerves canal; IX-XI, glossopharyngeal nerve canal and accessory nerves; XII, hypoglossal nerve canal. †Fossil taxon. Scale bars = 1cm.

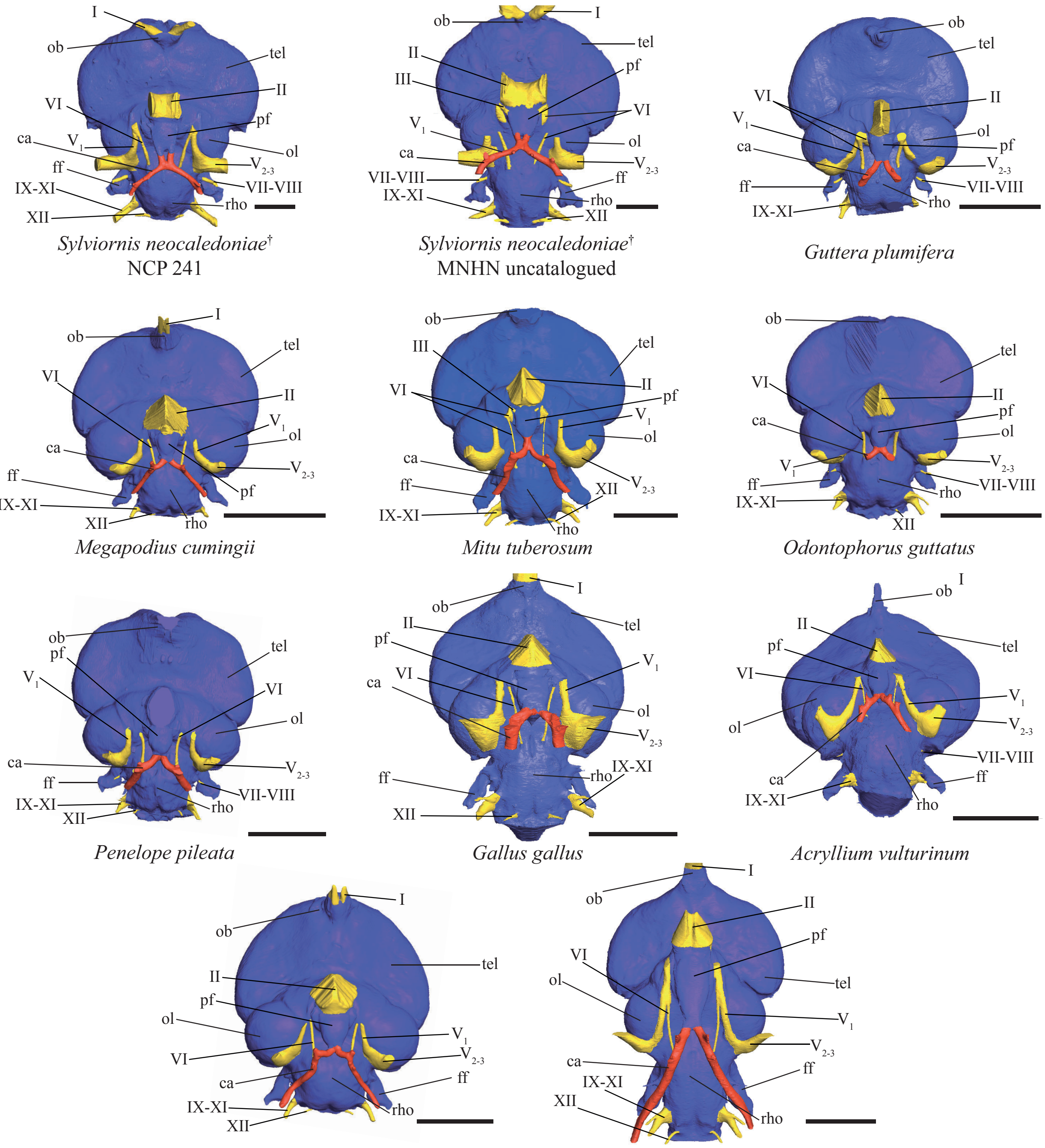

Figure S4. Ventral view of *S. neocaledoniae* and Galliformes endocasts. *Guttera plumifera* (Numididae, FSL-782); *Megapodius cumingii* (Megapodiidae, FSL-1.177); Mitu tuberosum (Cracidae, FSL-1.694); *Odontophorus guttatus* (Odontophoridae, FSL-1693); *Penelope pileata* (Cracidae, FSL-1.396); *Gallus gallus* (Phasianidae, NMS:Z.1931.43); *Acryllium vulturinum* (Numididae, FSL-1.391); *Alectura lathami* (Megapodiidae, MNHN-1923-2001); *Tetrao urogallus* (Phasianidae, MNHN-ME499). Abbreviations: ca, carotid artery canal; cer, optic lobe; pf; pituitary fossa; rho, rhombencephalon; tel, telencephalon; wslt, wulst; I, olfactory nerve canal; II, optic nerve canal; III, oculomotor nerve canal; V_1, ophthalmic nerve canal; V_{2-3}, maxillomandibular nerve canal; VI, abducens nerve canal; VII-VIII; facial and vestibulocochlear nerves canal; IX-XI, glossopharyngeal nerve canal and accessory nerves; XII, hypoglossal nerve canal. †Fossil taxon. Scale bars = 1cm.

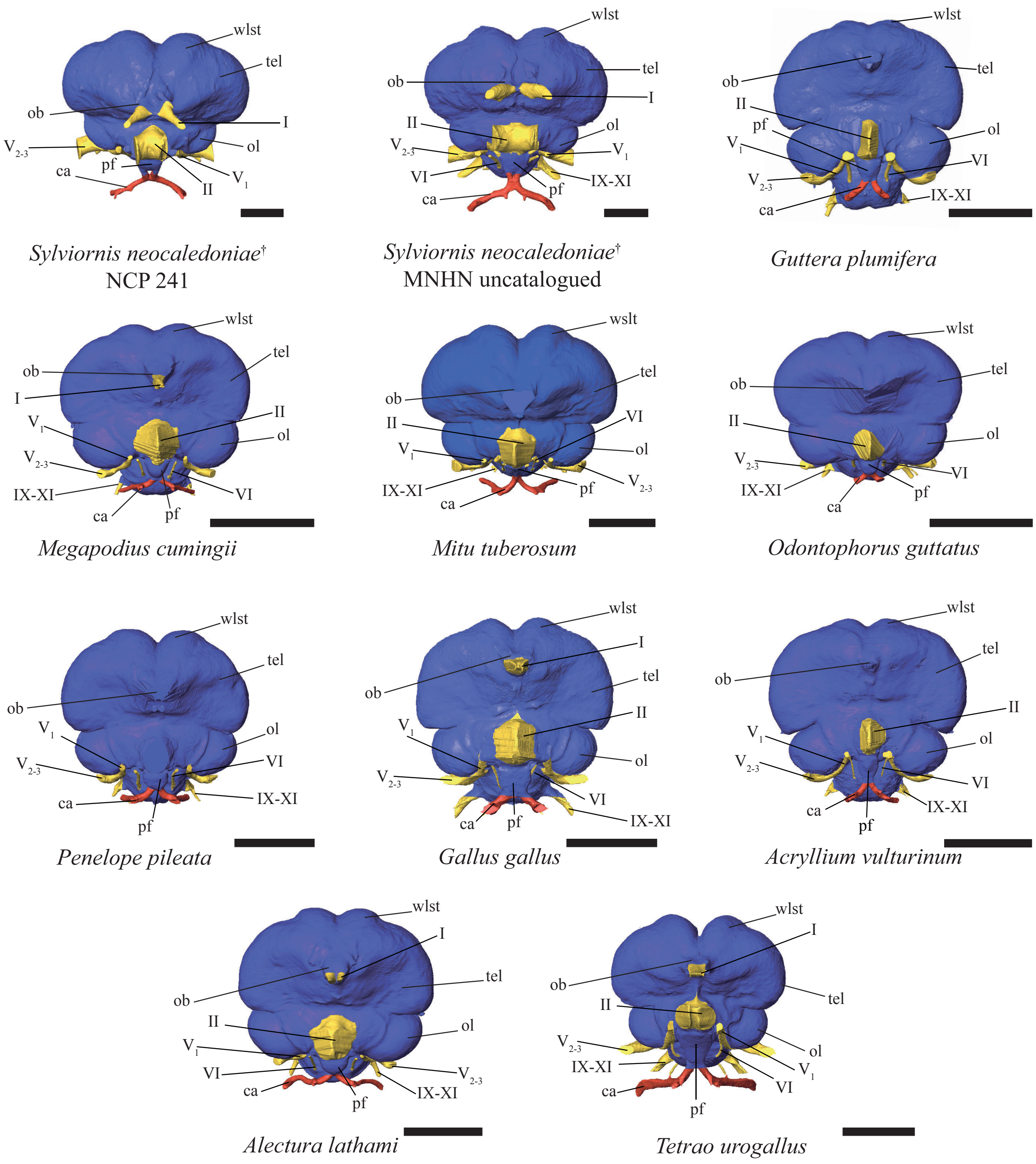

Figure S5. Rostral view of *S. neocaledoniae* and Galliformes endocasts. *Guttera plumifera* (Numididae, FSL-782); *Megapodius cumingii* (Megapodiidae, FSL-1.177); Mitu tuberosum (Cracidae, FSL-1.694); *Odontophorus guttatus* (Odontophoridae, FSL-1693); *Penelope pileata* (Cracidae, FSL-1.396); *Gallus gallus* (Phasianidae, NMS:Z.1931.43); *Acryllium vulturinum* (Numididae, FSL-1.391); *Alectura lathami* (Megapodiidae, MNHN-1923-2001); *Tetrao urogallus* (Phasianidae, MNHN-ME499). Abbreviations: ca, carotid artery canal; ob, olfactory bulb; ol, optic lobe; pf; pituitary fossa; tel, telencephalon; wslt, wulst; I, olfactory nerve canal; II, optic maxillomandibular nerve canal; VI, abducens nerve canal; VII-VIII; facial and vestibulocochlear nerves canal; IX-XI, glossopharyngeal nerve canal and accessory nerves; XII, hypoglossal nerve canal. †Fossil taxon. Scale bars = 1cm.

Inner ear

S. neocaledoniae have the semi-circular canals (ASC, LSC, PSC) slightly sinuous along with Cracidae (M. tuberosum and P. pilieata). Unlike the other galliforms which the semi-circular canals are relatively linear (Figure S7). The lateral semi-circular canal (LSC) and the posterior semi-circular canal (PSC) are more sinuous in *S. neocaledoniae* MNHN uncatalogued than in *S. neocaledoniae* NCP24. In *S. neocaledoniae*, the cochlear duct is slightly sinuous ventrally while the galliforms have a rectilinear cochlear duct (Figure S7). In *S. neocaledoniae*, the crus communis between the anterior and posterior semi-circular canals are located more dorsally on the anterior semi-circular canal (Figure S7). Overall, the inner ears of *S. neocaledoniae* are compressed laterally and rostrocaudally, just as in *M. tuberosum, O. Guttatus, P. pileate, A. lathami* and *T. urogallus* (Figure S7).

For all galliforms and *S. neocaledoniae*, the anterior semi-circular canal (ASC) is longer than the other two (Table S1), as shown by Hopkins (1906). *S. neocaledoniae* has a small anterior (ASC) and lateral (LSC) semi-circular canal. These values of ratio of length of the semi-circular channels on that of the hemisphere are part of the lowest. That for the posterior semi-circular canal (PSC) are average within galliforms (Figure S6a). The angles measured between the semi-circular canals are average in *S. neocaledoniae* compared to Galliformes. The angle between the lateral (LSC) and posterior (PSC) semi-circular canals is relatively small compared to those of the other galliforms (Figure S6b).

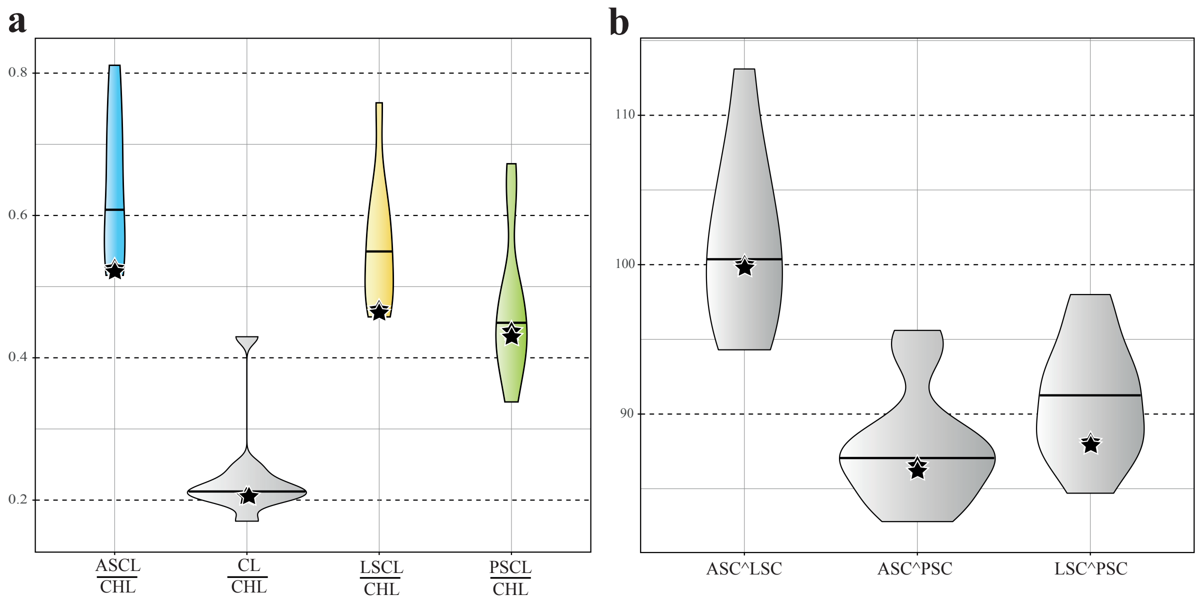

Figure S6. Violin plots showing the position of *S. neocaledoniae* (black star) relative to other Galliformes, with n=11, according to a series of ratios (a) and angles (b) of endosseous labyrinths. Heavy black line indicates the median. Abbreviations, ASCL, anterior semi-circular canal length; CHL, cerebral hemisphere length; C, cochlear duct length; LSCL, lateral semi-circular canal length; PSCL, posterior semi-circular canal length. The angles are given in degrees (^).

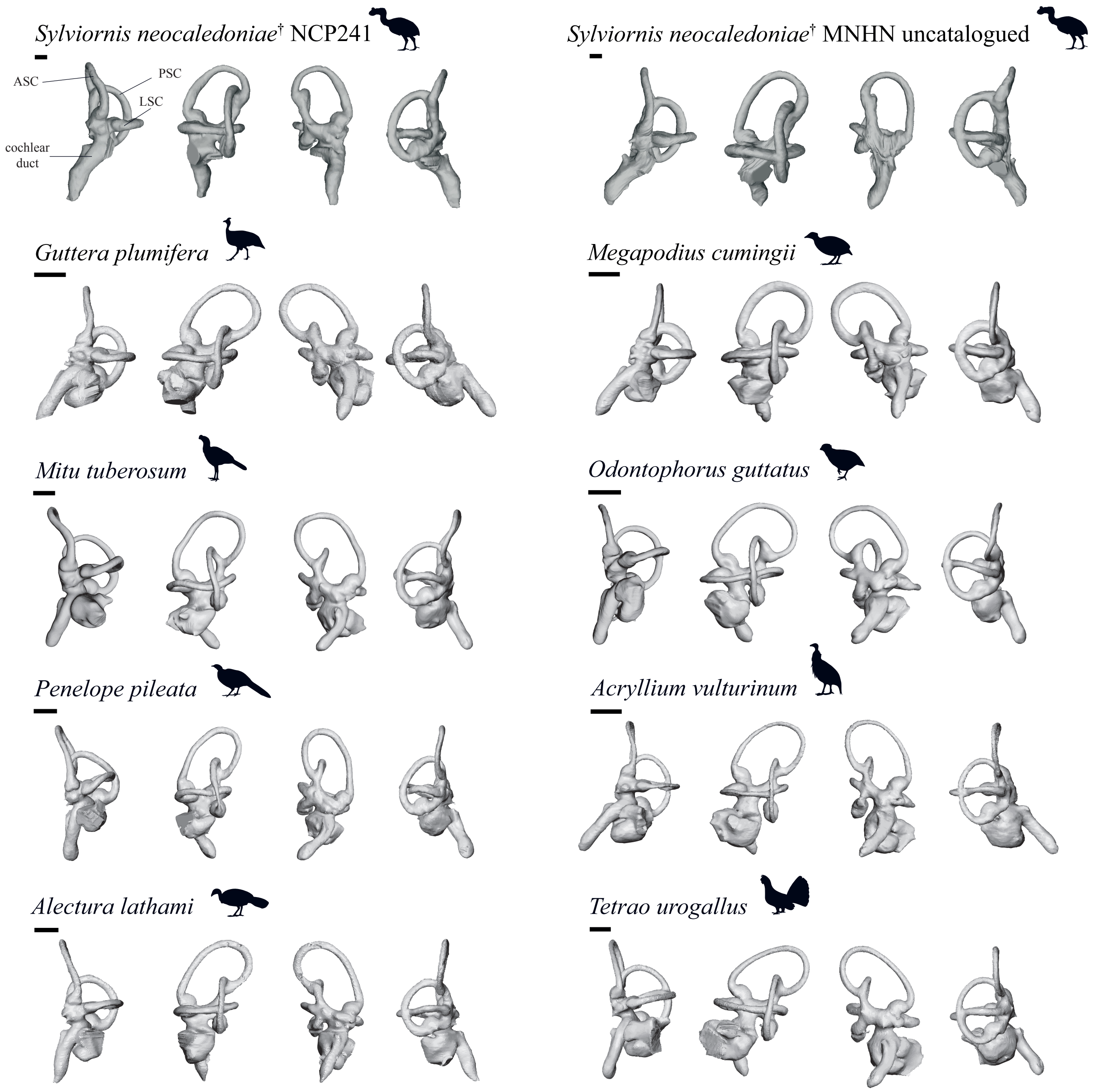

Figure S7. S. *neocaledoniae* and Galliformes endosseous labyrinths. *Guttera plumifera* (Numididae, FSL-782); *Megapodius cumingii* (Megapodidae, FSL-1.177); Mitu tuberosum (Cracidae, FSL-1.694); *Odontophorus guttatus* (Odontophoridae, FSL-1693); *Penelope pileata* (Cracidae, FSL-1.396); *Gallus gallus* (Phasianidae, NMS:Z.1931.43); *Acryllium vulturinum* (Numididae, FSL-1.391); *Alectura lathami* (Megapodidae, MNHN-1923-2001); *Tetrao urogallus* (Phasianidae, MNHN-ME499). Columns form left to right, sub-rostral view; sub-lateral view, face and back; sub-caudal view. Abbreviations, ASC, anterior semi-circular canal; LSC lateral semi-circular canal PSC, posterior semi-circular canal. †Fossil taxon. Scale bars = 2mm.

Endocast

Comparison of the two specimens of S. neocaledoniae

Both specimens have an endocast both well preserved and have a similar morphology. Thus S. neocaledoniae does not seem to show any individual morphological variation (Figures 1, S3, S4, S5). They exhibit a wulst, an olfactory bulb and a maxillomandibular nerve canal developed as well as a reduced optical lobe (Figure 1). *S. neocaledoniae* NCP241 presents measures higher than that of *S. neocaledoniae* MNHN uncatalogued. The specimen NCP 241 was probably a bigger adult.

Comparison with the other order of birds

S. neocaledoniae shows morphological differences with other bird orders. Here are some examples of morphological differences. In Anseriformes, Phoenicopteriformes, Podicepediformes and Gruiformes, the telencephalon is well developed rostrally, or even hypertrophied in *P. hochstetteri* (Gruiformes), unlike *S. neocaledoniae* (Figure S8, Figure 1). In ventral view, *O. hoazin* (Opistochomiformes) the hemisphere and optical lobe are distinct from each other, whereas in *S. neocaledoniae* they overlap (Figure S8, Figure S4). Unlike to S. neocaledoniae, *A. crecca* (Anseriformes), *P. roseus* (Phonicopteriformes), *P. nigricollis* (Podicepediformes) and *A. pygmaea* (Charadriiformes), have very pronounced cerebellum rostral flexion (Figure S8, Figure 1).

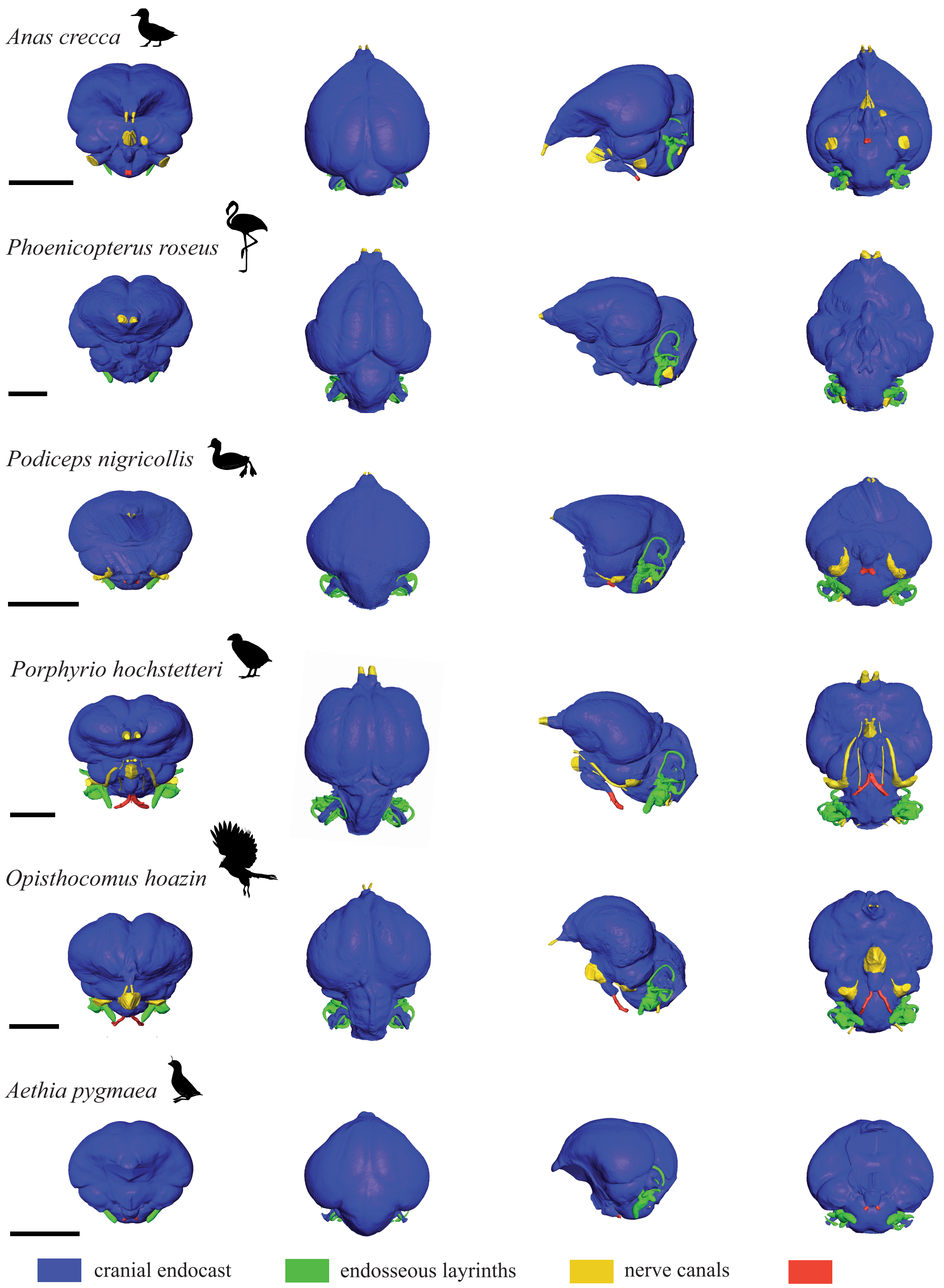

Figure S8. Endocasts *of other comparison orders. Anas crecca* (Ansériformes, ism:ism-aves:691539); *Phoenicopterus roseus* (Phoenicopteriformes, Uncatalogued – FSL) ; *Podiceps* nigricollii (Podicepediformes, ism:ism-aves:690281) *Porphyrio hochstetteri* (Gruiformes, MNHN uncatalogued)*; Aethia pygmaea* (Charadriiformes, ummz:birds:225610)*; Opisthocomus hoazin* (Opisthocomiformes, MNHN-1997-802). †Fossil taxon. Columns from left to right, rostral view; dorsal view; lateral view; ventral view. Scale bars = 1 cm.

Orbit and optic foramen size

Consistent with the size of its optical lobe, *S. neocaledoniae* shows a relatively small optic nerve, among the dataset set of Hall et al., 2009 (Figure S9). It has the diameter of the smallest optical foramen. Is associated with these two characteristics, an orbital diameter reduced in relation to the length of the skull. Indeed, *S. neocaledoniae* shows small orbit, the smallest, within galliforms, but also among the set of varied data studied in Hall et al. 2008 and Hall, 2008 (Figure S9). However, the very particular shape of the skull of *S. neocaledoniae*, flattened dorsoventrally and rostrocaudally, can induce a bias in the measurement of the ratio of the orbital diameter to the length of the skull. The combination of an optical lobe, optical nerves and reduced orbits, induce a reduced capacity in *S. neocaledoniae*.

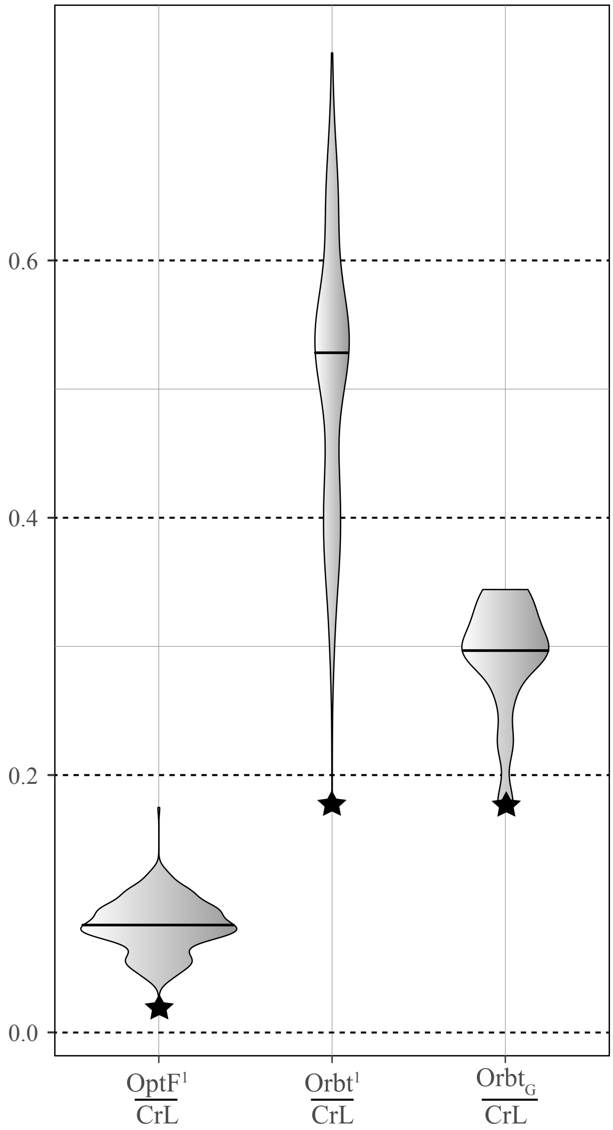

Figure S9. Violin plots showing the position of *S. neocaledoniae* (black star) relative to sampling of Hall et al., 2009 (n=321) and the other Galliformes of this study (n=11), according to a series of ratios. Heavy black line indicates the median. [1] data from Hall et al., 2009; $_G$ data from the other galliforms of this study. Abbreviations, CrL, cranium length; OptF, optic forman diameter; Orbt, orbit diameter.

Supplementary Figures

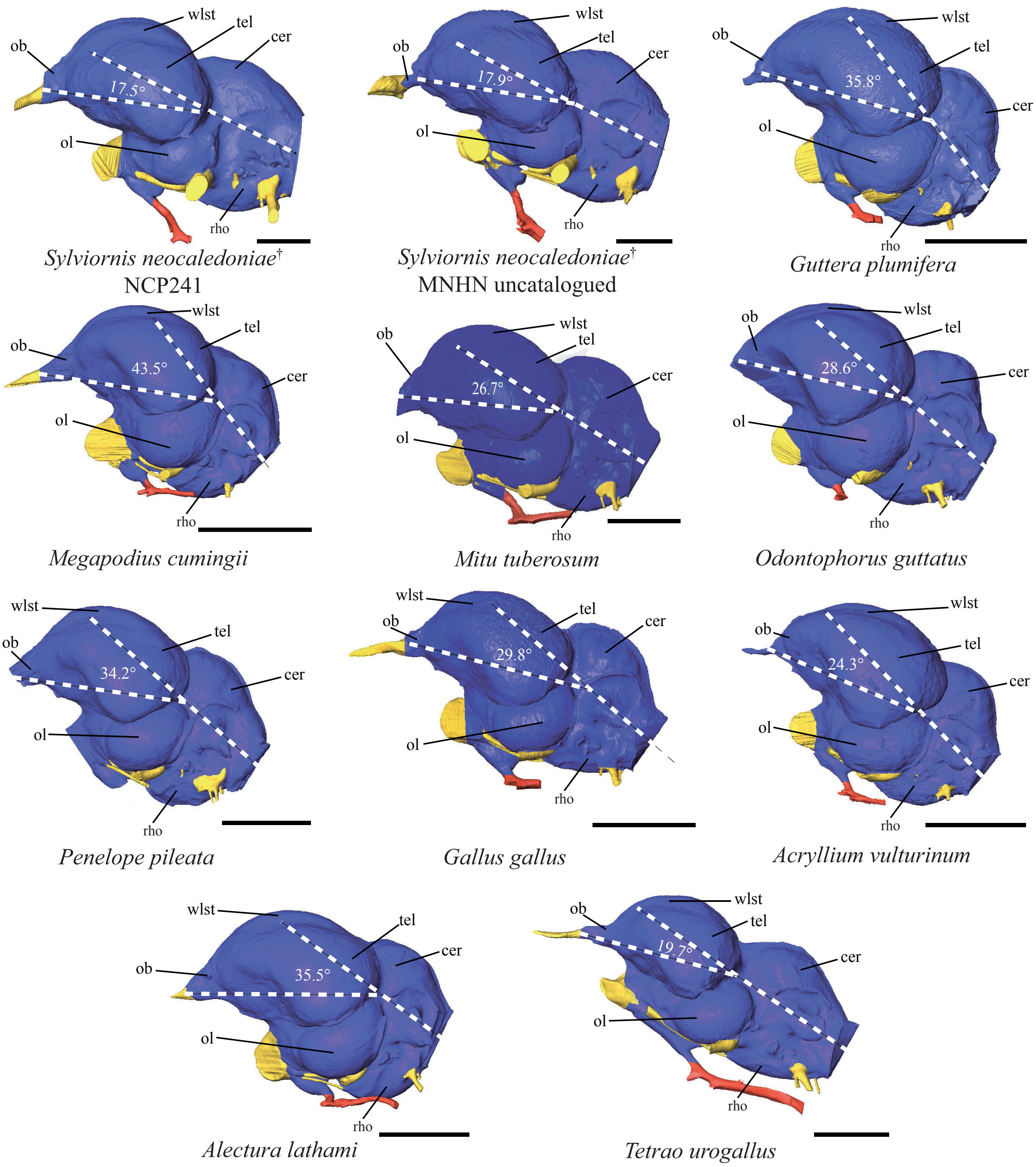

Figure S10. Orientation of *S. neocaledoniae* and Galliformes endocasts. Dashed lines indicate the path of the endocranial axis from the tip of the olfactory bulb to the midpoint of the isthmic constriction and thence to midpoint of the foramen magnum. Abbreviations, cer, cerebellum; ob, olfactory bulb; ol, optic lobe; rho, rhombencephalon; tel, telencephalon; wslt, wulst. †Fossil taxon. Lateral view. Scale bars = 1 cm.

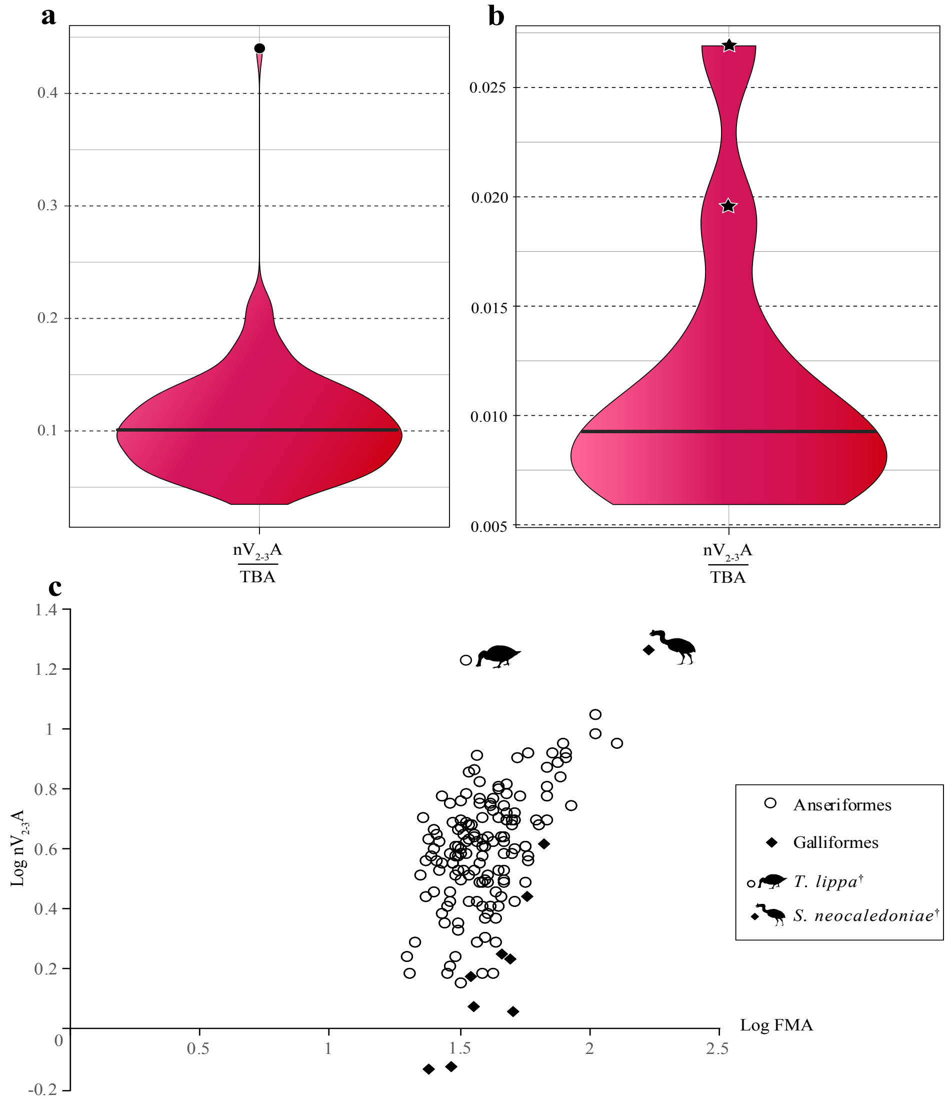

Figure S11. Violin plots showing the position of *S. neocaledoniae* (black star) relative to the other Galliformes, with n=11 (a), and the position of *T. lippa* (Anseriformes, back cercle) relative to the other Anseriformes, with n=150 (b) according to a ratio of maxillomandibular nerve canal. (c) Scatterplot of the maxillomandibular nerve canal cross-section area (log) against foramen magnum cross-section area (log),with n=161. Heavy black line indicates the median. Abbreviation, FMA, Foramen magnum cross-section area; $nV_{2\text{-}3}A$, maxillomandibular nerve canal cross-section area; TBA, total brain surface area. †Fossil taxon.

Supplementary Tables

Table S2_Scanning Parameters: Specimen information and CT Scanning parameters; †Fossil taxon; 1Natural History Museum, London provided access to these data originally appearing in Benson et al (2017: Comparative analysis of vestibular ecomorphology in birds, Journal of Anatomy), the collection of which was funded by Natural Environment Research Council (NERC) grants NE/H012176/1 and NE/E008380/1. The files were downloaded from -www.MorphoSource.org, Duke University; 2Rolland-Guillard et al., unpublished; 3University of Michigan Museum of Zoology provided access to these data, the collection of which was funded by oVert TCN; NSF DBI-1701714; NSF DBI-1701713. The files were downloaded from www.MorphoSource.org, Duke University; *Scanned for this study. Abbreviations: MNHN, Muséum National d'Histoire Naturelle, Paris, France; FSL, Faculté des Sciences de Lyon, Lyon, France.

Species	Common Name	Specimen Number	Scanner model	X-ray power (kv/mA)	Pixel dimension	#Slices	Voxel Size (mm^3)
Anas crecca[1,2]	Eurasian teal	ism:ism-aves:691539	Bruker Skyscan 1173	65/0,123	566 x 436	1173	0.068
Sylviornis neocaledoniae[†*]	Sylviornis	MNHN uncatalogued	GE Phoenix Nanotom 180 device	100/0,07	1776 x 1928	2086	0.0322
Sylviornis neocaledoniae[†*]	Sylviornis	MNHN-NCP 241	GE Phoenix Nanotom 180 device	100/0,07	4584 x 4264	3556	0.064
*Alectura lathami lathami**	Australian brushturkey	MNHN-1923-2001	GE Phoenix Nanotom 180 device	100/0,07	909 x 1029	654	0.0299
*Megapodius cumingii**	Philippine megapode	FSL-1.177	GE Phoenix Nanotom 180 device	100/0,07	1275 x 1491	840	0.0325
*Penelope pileata**	White-crested guan	FSL-1.396	GE Phoenix Nanotom 180 device	100/0,07	1083 x 999	1187	0.0325
*Mitu tuberosum**	Razor-billed curassow	FSL-1.694	GE Phoenix Nanotom 180 device	100/0,07	695 x 769	1090	0.0300
*Guttera plumifera**	Plumed guineafowl	FSL-782	GE Phoenix Nanotom 180 device	100/0,07	1071 x 2124	941	0.0299
*Acryllium vulturinum**	Pintade vulturine	FSL-1.391	GE Phoenix Nanotom 180 device	100/0,07	511 x 985	460	0.0325
*Odontophorus guttatus**	Spotted wood quail	FSL-1693	GE Phoenix Nanotom 180 device	100/0,07	1482 x 1227	891	0.0299
*Gallus gallus**	Chicken	NMS:Z.1931.43	Nikon XTH 225 ST	100/0,07	685 x 659	346	0.055
*Tetrao urogallus**	Western capercaillie	MNHN-ME499	GE Phoenix Nanotom 180 device	100/0,07	873 x 981	945	0.0299
Phoenicopterus roseus[2]	Greater flamingo	Uncatalogued - FSL	GE Phoenix Nanotom 180 device	100/0,07	1474x1827	2302	0.030
Podiceps nigricollii[1,2]	Black-necked grebe	ism:ism-aves:690281	Bruker Skyscan 1173	65/0,123	633x435	999	0.055
Porphyrio hochstetteri[*]	South Island Takahe	MNHN uncatalogued	GE Phoenix Nanotom 180 device	100/0,07	914 x 1270	1646	0.0325
Aethia pygmaea[2,3]	Whiskered auklet	ummz:birds:225610	Nikon XTH 225 ST	85/0,2	508 x 382	545	0.079
Opisthocomus hoazin[2]	Hoatzin	MNHN-1997-802	GE Phoenix Nanotom 180 device	100/0,07	843 x 636	510	0.0325

Table S3_Brain ratios data for galliforms studied and big birds of other studies. CHR, ratio of cerebral hemisphere area to total brain surface area; CrbR, ratio of cerebellum area to total brain surface area; OptR, ratio of optic lobe area to total brain surface area; WR, ratio of wulst area to total brain surface area, OR, ratio of olfactory bulb length to cerebral hemisphere length; nV2-3R, ratio of section of maxillomandibular nerve area to total brain area. [1]Torres & Clarke, 2018; [2]Early et al., 2019; [3]Corfield et al., 2016; [4]Iwaniuk et al., 2008; * this study; †Fossil taxon.

Species	Common Name	CHR	CrbR	OptR	WR	OR	$nV_{2-3}R$
Sylviornis neocaledoniae† MNHN uncatalogued*	sylviornis	0.17	0.08	0.04	0.05	0.19	0.0019
Sylviornis neocaledoniae† NCP 241*	sylviornis	0.16	0.07	0.04	0.05	0.20	0.0027
*Tetrao urogallus**	western capercaillie	0.13	0.08	0.06	0.05	0.18	0.001
*Gallus gallus**	chicken	0.15	0.07	0.06	0.03	0.12	0.001
*Alectura lathami**	australian brushturkey	0.17	0.07	0.07	0.03	0.15	0.0006
*Megapodius cumingii**	philippine megapode whit-	0.17	0.08	0.07	0.04	0.14	0.0006
*Penelope pileata**	crested guan	0.16	0.06	0.06	0.04	0.16	0.0009
*Mitu tuberosum**	razor-billed curassow	0.18	0.06	0.06	0.04	0.15	0.0014
*Odontophorus guttatus**	spotted wood quail plumed	0.17	0.06	0.08	0.03	0.19	0.0007
*Guttera plumifera**	guineafowl vulturine	0.18	0.07	0.07	0.02	0.16	0.0007
*Acryllium vulturinum**	guineafowl southern	0.18	0.06	0.06	0.03	0.17	0.009
Casuarius casuarius[1]	cassowary			0.04		0.27	
Dromaius novaehollandiae[1]	emu			0.04		0.27	
Aepyornis maximus†1	elephant bird			0.02		0.26	
Pachyornis elephantopus†1	heavy-footed moa			0.04		0.21	
Struthio camelus[1]	common ostrich			0.07		0.18	
Rhea americana[1]	greater rhea			0.09		0.22	
Dinornis robustus†2	south island giant moa			0.02	0.18	0.023	
Dromaius novaehollandiae[3]	emu			0.08	0.16	0.08	
Llallawavis scagliai†2	magnificent bird of			0.08	0.19	0.08	
Meleagris gallopavo[4]	scaglia wild turkey			0.14	0.09	0.14	
Paraptenodytes antarcticus†2				0.08	0.15	0.08	
Psilopterus lemoinei†2				0.09	0.17	0.09	
Rhea americana[4]	greater rhea			0.11	0.11	0.11	

References

1. Balanoff, A.M., Bever, G.S., Colbert, M.W., Clarke, J.A., Field, D.J., Gignac, P.M., Ksepka, D.T., Ridgely, R.C., Smith, N.A., Torres, C.R., Walsh, S. and Witmer, L.M. (2016). Best practices for digitally constructing endocranial casts: examples from birds and their dinosaurian relatives. *Journal of Anatomy*, 229(2), 173-190.
2. Baumel, J. J. (1993). Handbook of avian anatomy: nomina anatomica avium. Publications of the Nuttall Ornithological Club (USA). no. 23.
3. Benson, R. B., Starmer-Jones, E., Close, R. A., & Walsh, S. A. (2017). Comparative analysis of vestibular ecomorphology in birds. *Journal of Anatomy*, 231(6), 990-1018.
4. Cignoni, P., Callieri, M., Corsini, M., Dellepiane, M., Ganovelli, F., & Ranzuglia, G. (2008, July). Meshlab: an open-source mesh processing tool. In *Eurographics Italian chapter conference*, 2008, 129-136.
5. Corfield, J. R., Kolominsky, J., Craciun, I., Mulvany-Robbins, B. E., & Wylie, D. R. Is cerebellar architecture shaped by sensory ecology in the New Zealand Kiwi (*Apteryx mantelli*)?. *Brain, Behavior and Evolution*, 87(2), 88-104 (2016).
6. Early, C. M. (2019). Quantitative Assessments of Avian Endocasts as Tools for Inferring Neuroanatomical Traits and Potential Functional Capabilities (Doctoral dissertation, Ohio University).
7. Early, C. M., Ridgely, R. C., & Witmer, L. M. (2020). Beyond endocasts: using predicted brain-structure volumes of extinct birds to assess neuroanatomical and behavioral inferences. *Diversity*, *12*(1), 34.
8. Hall, M. I. (2008). The anatomical relationships between the avian eye, orbit and sclerotic ring: implications for inferring activity patterns in extinct birds. Journal of Anatomy, 212(6), 781-794.

9. Hall, M. I., Iwaniuk, A. N., & Gutiérrez-Ibáñez, C. (2009). Optic foramen morphology and activity pattern in birds. *The Anatomical Record: Advances in Integrative Anatomy and Evolutionary Biology*, 292(11), 1827-1845.
10. Handley, W. D., & Worthy, T. H. (2021). Endocranial Anatomy of the Giant Extinct Australian Mihirung Birds (Aves, Dromornithidae). *Diversity*, 13(3), 124.
11. Hopkins, M. A. (1906). On the relative dimensions of the osseous semicircular canals of birds. *The Biological Bulletin*, 11(5), 253-264.
12. Kimball, R. T., Oliveros, C. H., Wang, N., White, N. D., Barker, F. K., Field, D. J., Ksepka, D. T., Chesser, R. T., Moyle, R. G., Braun, M. J., Brunfield, R. T., Faircloth, B. C., Smith, B. T. & Braun, E. L. (2019). A phylogenomic supertree of birds. *Diversity*, 11(7), 109.
13. Kuhl, H., Frankl-Vilches, C., Bakker, A., Mayr, G., Nikolaus, G., Boerno, S. T., Klages, S., Timmermann, B. & Gahr, M. (2021). An Unbiased Molecular Approach Using 3′-UTRs Resolves the Avian Family-Level Tree of Life. *Molecular biology and evolution*, 38(1), 108-127.
14. Torres, C. R., & Clarke, J. A. (2018). Nocturnal giants: evolution of the sensory ecology in elephant birds and other palaeognaths inferred from digital brain reconstructions. *Proceedings of the Royal Society B*, 285(1890), 20181540.
